# Cetuximab-induced natural killer cell cytotoxicity in head and neck squamous cell carcinoma cell lines: investigation of the role of cetuximab sensitivity and HPV status

**DOI:** 10.1038/s41416-020-0934-3

**Published:** 2020-06-16

**Authors:** Hasan Baysal, Ines De Pauw, Hannah Zaryouh, Jorrit De Waele, Marc Peeters, Patrick Pauwels, Jan Baptist Vermorken, Evelien Smits, Filip Lardon, Julie Jacobs, An Wouters

**Affiliations:** 1 Center for Oncological Research (CORE), University of Antwerp, Antwerp, Belgium; 2 Department of Oncology, Antwerp University Hospital, Antwerp, Belgium; 3 Department of Pathology, Antwerp University Hospital, Antwerp, Belgium; 4 Center for Cell Therapy and Regenerative Medicine, Antwerp University Hospital, Antwerp, Belgium

**Keywords:** Head and neck cancer, Innate lymphoid cells, Targeted therapies, Cancer therapeutic resistance

## Abstract

**Background:**

The epidermal growth factor receptor (EGFR) is overexpressed by 80–90% of squamous cell carcinoma of head and neck (HNSCC). In addition to inhibiting EGFR signal transduction, cetuximab, a monoclonal antibody targeting EGFR can also bind to fragment crystallisable domain of immunoglobulins G1 present on natural killer (NK), causing antibody-dependent cellular cytotoxicity (ADCC). However, presence of cetuximab resistance limits effective clinical management of HNSCC.

**Methods:**

In this study, differences in induction of ADCC were investigated in a panel of ten HNSCC cell lines. Tumour cells were co-cultured with NK cells and monitored using the xCELLigence RTCA.

**Results:**

While ADCC was not influenced by HPV status, hypoxia and cetuximab resistance did affect ADCC differentially. Intrinsic cetuximab-resistant cell lines showed an increased ADCC induction, whereas exposure to hypoxia reduced ADCC. Baseline EGFR expression was not correlated with ADCC. In contrast, EGFR internalisation following cetuximab treatment was positively correlated with ADCC.

**Conclusion:**

These findings support the possibility that resistance against cetuximab can be overcome by NK cell-based immune reactions. As such, it provides an incentive to combine cetuximab with immunotherapeutic approaches, thereby possibly enhancing the anti-tumoural immune responses and achieving greater clinical effectiveness of EGFR-targeting agents.

## Background

Head and neck squamous cell carcinoma (HNSCC) remains the sixth most common cancer type with incidence rates reaching nearly 750,000 cases/year.^1^ Besides the main risk factors such as tobacco and alcohol consumption, infection with high-risk types of human papillomavirus (HPV) also plays an important role.^2^ Clinical data show HPV-positive patients have a more favourable prognosis with respect to recurrence and survival.^3^ However, evidence regarding the predictive role of HPV in treatment of HNSCC is largely missing. Despite advances in conventional treatments, 5-year survival for metastatic HNSCC has remained only 40%.^4^ Therefore, innovative therapeutic strategies are necessary to increase survival outcomes and reduce substantial toxicities of conventional treatments.

Both targeted therapies and immunotherapies are now at the forefront of personalised cancer medicine. Aberrant signalling of the epidermal growth factor receptor (EGFR) plays an integral role in the tumorigenesis of multiple cancer types, including HNSCC.^5,6^ EGFR belongs to the human receptor tyrosine kinase family and signalling of EGFR regulates cellular processes involved in anti-apoptotic signalling, proliferation, differentiation, migration and angiogenesis.^7^ 80–90% of HNSCC show overexpression of EGFR, making it a compelling drug target for inhibition.^8^ Two classes of EGFR inhibitors have been developed, namely the tyrosine kinase inhibitors (TKIs), including erlotinib and gefitinib, as well as monoclonal antibodies (mAbs) such as cetuximab and panitumumab.^9^ Nonetheless, although increased expression of EGFR is a negative prognostic factor and has been linked to poor prognosis in HNSCC patients,^10^ it has no clear predictive role for treatment with EGFR-targeted therapeutic agents.^11^

Cetuximab is a chimeric EGFR-blocking IgG1 mAb that binds the extracellular domain of EGFR, thereby preventing receptor activation and downstream signalling, as well as inducing internalisation.^12^ Clinical data of cetuximab showed improved overall survival (OS) in both locally advanced and recurrent/metastatic settings, when combined with radiation (RT) versus RT alone (median OS 49 vs. 29 months) or combined with chemotherapy (CT) versus CT alone (median OS 10.1 vs. 7.4 months), respectively.^5,13^ These results led to FDA approval of cetuximab for the treatment of locally advanced- as well as recurrent and metastatic- HNSCC. However, issues such as tumour heterogeneity and multiple resistance mechanisms have limited the observed response rates (RR) to only 13% for cetuximab monotherapy and 36% in combination with CT (compared to 20% RR for CT alone).^13,14^ Interestingly, besides its direct receptor blocking ability, both preclinical and clinical evidence have shown that cetuximab also induces immunologic anti-tumour effects.^15,16^ Cetuximab consists of an IgG1 backbone, through which it can bind CD16 fragment crystallisable (Fc) receptors located on natural killer (NK) cells, macrophages and granulocytes, of which NK cells have been proven to be the most potent effectors.^17^ Binding of the IgG1-Fc part of cetuximab to CD16 on NK cells triggers cytolytic activity called antibody-dependent cellular cytotoxicity (ADCC), which is predominantly mediated by perforin and granzymes.^16^ Furthermore, cetuximab has been shown to enhance cross-priming of cytotoxic T-lymphocytes via professional antigen-presenting cells, such as dendritic cells,^18^ mainly through induction of immunogenic cell death of tumour cells.^19^ These results confirmed the important immune-related mechanism of action of cetuximab, in addition to its receptor blocking effects.

Importantly, the tumour microenvironment (TME) plays a crucial role in the effectiveness of cancer therapies, e.g. through metabolic changes within the tumour cells. In this regard, we have previously shown that activation of hypoxia‐inducible factor (HIF) signalling is closely linked with EGFR-signalling, as HIFs are key survival factors under hypoxic conditions.^20^ Furthermore, changes in oxygen level have been previously shown to affect NK cell activity as well.^21^ Together with the findings that hypoxia is frequently seen in most solid tumours,^22^ it is of crucial importance to take the hypoxic tumour microenvironment into account.

Overall, we aim at investigating whether the capacity of cetuximab to induce ADCC depends on cetuximab sensitivity (sensitive, intrinsic/acquired resistant) and/or HPV status in a broad panel of HNSCC cell lines. Furthermore, as we previously showed that EGFR expression within our panel of cell lines was highly cell line-dependent and was significantly altered under hypoxia,^23^ we also determined whether ADCC was correlated with EGFR expression. Lastly, we investigated whether differences in ADCC could be associated with differences in EGFR internalisation (Fig. S1).

## Methods

### Cell lines and cell culture

Ten human HNSCC cell lines with different sensitivities to cetuximab and HPV status were included. UM‐SCC‐104 and UM-SCC-090 were obtained from American Type Culture Collection (ATCC, Rockville, MD, USA) and Cal‐27 was obtained from Merck Millipore (SA/NV, Overijse, Belgium). SC263 and SQD9 were kindly provided by prof. Sandra Nuyts (University Hospital Leuven, Leuven, Belgium), and LICR‐HN1 and SCC22b were kindly provided by prof. Olivier De Wever (Laboratory of Experimental Cancer Research, Ghent University Hospital, Ghent, Belgium). 93‐VU-147‐T was kindly provided by Dr. Josephine Dorsman (VU University Medical Center, Amsterdam, The Netherlands). Acquired cetuximab-resistant (Acq_Res_) variants of the originally cetuximab-sensitive (Cet_Sen_) parental SCC22b and SC263 cell lines were generated as described previously (suffix R).^24^ As a control for vehicle exposure and an increased culture period, cells were also exposed to the vehicle control, i.e. PBS (suffix PBS). All other cell lines were previously shown to be intrinsically cetuximab-resistant (Int_Res_).^23^ To confirm permanent resistance, dose-response was re-assessed after culturing resistant cell lines for 6 weeks in absence of cetuximab.

All cell lines were HPV-negative, except for the HPV-positive 93‐VU-147‐T, UM‐SCC‐104 and UM-SCC-090 cell lines. All HNSCC cell lines were cultured in DMEM (Gibco, 10938025), except for the UM-SCC-090 cells, which were cultured in MEM (Gibco, 11090081). Base media were supplemented with 10% foetal bovine serum (Gibco, 10270106), 1% penicillin/streptomycin (Gibco, 15140122), and 2 mM l‐glutamine (Gibco, 25030081). MEM was additionally supplemented with 1% non-essential amino acids (Gibco, 11140035). Cells were grown as monolayers and maintained in exponential growth in 5% CO_2_/95% air in a humidified incubator at 37 °C. All cell lines were confirmed free of mycoplasma infection through regular testing (Lonza, LT07–118). Additionally, all cell line identities were validated through short tandem repeat profiling. Hypoxic conditions (1% O_2_) were achieved by the use of a Bactron IV anaerobic chamber (Shel Lab), as described previously.^25^ Hypoxic conditions were initiated immediately after addition of the treatment.

### Human NK cells

Peripheral blood mononuclear cells (PBMC) were isolated from buffy coats of healthy donors (Belgian Red Cross—Blood Transfusion Center, Mechelen, Belgium) using Lymphoprep density gradient centrifugation (STEMCELL technologies, 07851) and frozen in FBS containing 10% dimethyl sulfoxide (Sigma, D2650) in liquid nitrogen (Nippon Gasses). For experiments, PBMC were thawed one day in advance in RPMI (Gibco, 52400) supplemented with 10% FBS, [2 mM] L-glutamine, [1 mM] sodium pyruvate (Gibco, 11360039), 1% penicillin/streptomycin, and [1.5KU/ml] DNase I (Sigma, D4263). CD56^+^CD3^−^ NK cells were obtained from PBMCs using a human NK cell isolation kit (Miltenyi; 130–092–657) according to the manufacturer's instructions. Purity of NK cell isolation was assessed on the Cytoflex flow cytometer (Beckman Coulter) using CD3-FITC (BD, 561806) and CD56-PE (BD, 555516) labelled mAbs. A cut-off of 80% CD56^+^CD3^−^ NK cells was used to define an adequate isolation for further experiments. NK cells were resuspended in DMEM or MEM for further experiments.

Retrovirally transfected NK92 cells, expressing CD16, were kindly provided by Dr. Nadia Mensali (Oslo University Hospital, Norway) and maintained in X-vivo 10 medium supplemented with 5% human serum and [500U/ml] interleukin-2. The initial population of NK92 cells consisted of 86% CD16^+^ cells and these cells were subsequently sorted into CD16^+^ and CD16^−^ populations using anti-human CD16 PE-conjugated mAb (Immunotools, 21279164) on the FACSAria II (BD biosciences).

### Growth inhibition assay

The growth inhibitory effects of cetuximab were assessed using the colorimetric sulforhodamine B (SRB) assay as described previously.^26^ Cells were harvested using 0.05% Trypsin-EDTA (Gibco, 25300062) and counted using the TC20^TM^ automated cell counter (Bio-Rad). Cells were seeded in a 96-well plate and left for overnight incubation. The following day, cetuximab [0–15 µg/ml] was added. After 48 h, cells were fixed with 10% trichloric acid and stained with 0.01% SRB followed by measurement of the optical density at 540 nm using the iMark microplate reader (Biorad).

### ADCC assay

Cytotoxicity studies were performed using gold-coated 16-well plates (E-plate16) for the xCELLigence Real-Time Cell Analysis (RTCA) (Roche Diagnostics, 00380601050), as described previously.^27^ Seeding density was optimised for each HNSCC cell line to ensure continuous growth until the end of the assay. Briefly, cells were harvested, counted and background impedance of the E-plate16 was measured before seeding of cells. After overnight incubation, cetuximab [2 µg/ml] or human immunoglobulin G1 kappa (IgG1κ, [2 µg/ml]) (Sigma, 5154), as an isotype control was added. Additionally, either cell-free medium or isolated NK cells were added to each well at an effector (NK cells) to target (tumour cells) (E:T) ratio of 5:1. The impedance was followed up by automated measurement every 15 min starting from cell seeding and ending 48 h after treatment with cetuximab both under normoxic and hypoxic conditions. Measurement of the impedance was expressed as Cell Index (CI). Each condition was performed in dublo.

### EGFR expression

Previous experiments determined baseline EGFR expression in our panel of HNSCC cell lines through flow cytometry.^23^ Briefly, cells were harvested, counted and stained with an EGFR PE‐conjugated antibody (10 µl/10^6^ cells; FAB10951P; R&D Systems) or an isotype (rat IgG2A; 10 μL/10^6^ cells; IC006P; R&D Systems), as a negative control. Live/Dead Fixable Far‐Red Dead Cell Stain Kit (Thermo Fisher) was used to exclude dead cells. Samples were fixed in 4% formaldehyde for 10 min under normoxic and hypoxic conditions. Samples were measured on the FACSAccuri (BD Biosciences) and analysed through FLOWJO version 10.1 (TreeStar Inc.) The percentage positive cells (overton) and mean fluorescence intensities (ΔMFI) were calculated by subtracting the signal of the isotype from the signal of the sample.

### EGFR internalisation

All HNSCC cell lines were seeded in 96-well plates and left overnight to adhere until a confluence of 40–50% was reached the next day. Cetuximab and an IgG1κ isotype were labelled with IncuCyte® Human FabFluor-pH Red Antibody Labeling Reagent (Essen Bioscience, 4722) at a molar ratio of 1:3 (test Ab : labelling Fab), according to the manufacturer’s protocol. The intensity of the FabFluor reagent is pH-dependent. As such, a fluorogenic signal is observed as the Fab-Ab complex is internalised and processed via acidic lysosomes and endosomes. Ab-Fab mixes were added to cells at a concentration of [2 µg/mL] and plates were placed into the IncuCyte ZOOM Live-Cell Analysis System (Essen BioScience) inside an incubator at 37 °C and 5% CO_2_. Phase and red fluorescence channels were acquired with a 10× objective immediately after addition of Ab-Fab mix to cells and followed up for 24 h. Each condition was performed in triplo, with four images being captured and analysed per well, per time point. The total red object area (µm^2^/well) was quantified for each time point and normalised to the cell confluence (%) using the IncuCyte ZOOM software version 2018B.

### Statistical analysis

At least three independent experiments were performed for all experiments, with NK cells isolated from three different donors. Moreover, all conditions were plated at least in duplo. Results are presented as mean ± standard error of the mean (SEM). A linear mixed model was used to assess the influence of cetuximab sensitivity, HPV- and oxygenation-status on cetuximab-mediated ADCC and EGFR internalisation. A stepwise backward approach was applied, starting from a model with all fixed and random effects and their interactions. If the interaction term was not significant, a model with only the main effects was fitted. Significance between conditions was assessed using Tukey's multiple comparison. The possible correlation between EGFR expression, internalisation and cetuximab-mediated ADCC was evaluated through a pairwise Spearman’s rank correlation. All statistical analyses were conducted using SPSS statistics 25 (IBM) and JMP Pro 14 (SAS). *P*-values < 0.05 were considered statistically significant.

## Results

### Direct growth inhibitory effect of cetuximab on HNSCC cell lines

**Fig. 1:**
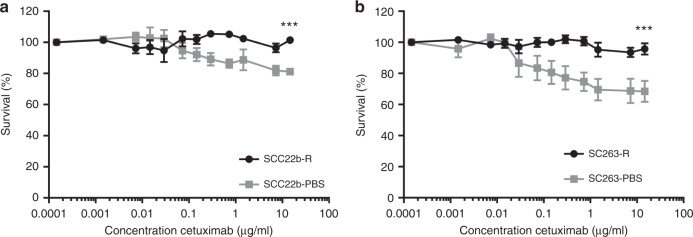
Cetuximab induces minimal growth inhibitory effect on HNSCC cell lines after 48-h treatment. Dose-response curves of the SCC22b-PBS, SCC22b-R (**a**); SC263-PBS and SC263-R cell lines (**b**) were evaluated using the SRB assay. Graphs show mean survival ± SEM of at least three individual experiments. Statistical significance between cell lines was tested using a linear mixed model. ***p < 0.001.

We previously identified the sensitivity of our panel of ten HNSCC cell lines to cetuximab treatment for 168 h using the SRB assay,^23^ thereby classifying cell lines in Cet_Sen_, Int_Res_ and Acq_Res_ cell lines. To study cytotoxic effects of short-term exposure to cetuximab for 48 h (time point used in ADCC assays), we determined dose-response curves for the Cet_Sen_ SCC22b-PBS, SC263-PBS cell lines and the Acq_Res_ SCC22b-R, SC263-R cell lines (Fig. 1). Following a 48-h treatment period, none of the cell lines reached a 50% decrease in cell survival even with the highest concentration of cetuximab [15 µg/ml] (SCC22b-R: 101.42 ± 1.64%; SCC22b-PBS: 81.13 ± 1.99%; SC263-R: 95.75 ± 3.65%; SC263-PBS: 68.4 ± 6.74%). However, the Acq_Res_ cell lines were statistically less sensitive (*p* < 0.001) to growth inhibition by cetuximab compared to the corresponding isogenic Cet_Sen_ cell lines. All other HNSCC cell lines were intrinsically resistant to cetuximab treatment (data not shown).

### NK92 cells induce natural cytotoxicity towards HNSCC cell lines

**Fig. 2:**
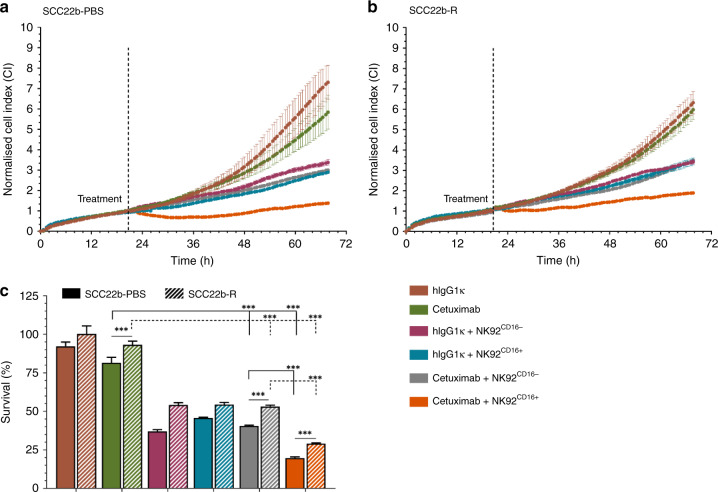
Cetuximab induces NK-cell mediated killing through ADCC in HNSCC cell lines. Cell index (CI) of the SCC22b-PBS (**a**) and SCC22b-R (**b**) was measured using the xCELLigence RTCA. Following overnight incubation, HNSCC cell lines were treated with an isotype control (hIgG1κ) or cetuximab [2 µg/ml], with or without NK92 cells (effector to target (E:T) ratio of 5:1), and CI was monitored for 48-h post-treatment (**c**). Both CD16^+^ and CD16^−^ NK92 cells were used. CI was normalised to the time point were treatments (isotype control, cetuximab and/or NK92 cells) were added. Graphs represent mean ± SEM for each time point of follow up. Statistical significance between cell lines was tested using a linear mixed model with Tukey’s pairwise comparison. NS not significant; ***p < 0.001.

Next, using the xCELLigence RTCA system, we identified cetuximab-induced ADCC in two HNSCC cell lines (SCC22b-PBS and SCC22b-R) in co-culture with CD16-positive or CD16-negative NK92 cells (NK92^CD16-^, NK92^CD16+^, respectively) (E:T = 5:1) to prove the attribution of ADCC in this setting(Fig. S2). Incubation with cetuximab [2 µg/ml] for 48-h reduced survival of the SCC22b-PBS to 81.53 ± 3.53% compared to isotype control [2 µg/ml] (*p* < 0.001) (Fig. 2a). Cetuximab treatment of the SCC22b-R cells only reduced cell survival to 93.19 ± 2.36% (*p* = 0.305) (Fig. 2b). Meanwhile, co-culture of HNSCC cell lines with NK92^CD16-^ cells demonstrated a significant decrease in cell growth (SCC22b-PBS: 36.99 ± 1.11%, *p* < 0.001; SCC22b-R: 54.22 ± 1.41%, *p* < 0.001) compared to cetuximab alone. Similar decreases were observed with addition of NK92^CD16+^ (SCC22b-PBS: 45.73 ± 0.49%, *p* < 0.001; SCC22b-R: 54.44 ± 1.39%, *p* < 0.001). However, addition of cetuximab and NK92^CD16-^ cells to either SCC22b-PBS or SCC22b-R HNSCC cells did not result in a significant difference in cell survival compared to addition of NK92^CD16-^ alone (SCC22b-PBS: 40.60 ± 0.41%, SCC22b-R: 53.16 ± 0.80%, *p* ≥ 0.681) (Fig. 2c). In contrast, addition of cetuximab and NK92^CD16+^ cells to HNSCC cell lines, clearly increased tumour cell killing, as shown by a further decrease in survival in both the SCC22b-PBS (19.71 ± 0.75%, *p* < 0.001) and SCC22b-R (29.07 ± 0.47%, *p* < 0.001) cell lines (Fig. 2c), compared to addition of NK92^CD16+^ alone. Lastly, the SCC22b-R cell line, in addition to being less sensitive to cetuximab treatment, was significantly less sensitive to NK92^CD16+^-mediated ADCC, compared to the SCC22b-PBS cell line (*p* < 0.001). These results confirm the minimal growth inhibitory effect of cetuximab after 48-h treatment and show increased NK-cell killing following cetuximab treatment can be attributed to interaction between cetuximab and CD16, inducing ADCC. Finally, these results also show that ADCC can potentially overcome cetuximab resistance.

### Real-time follow up of cetuximab-mediated ADCC against HNSCC cell lines

**Fig. 3:**
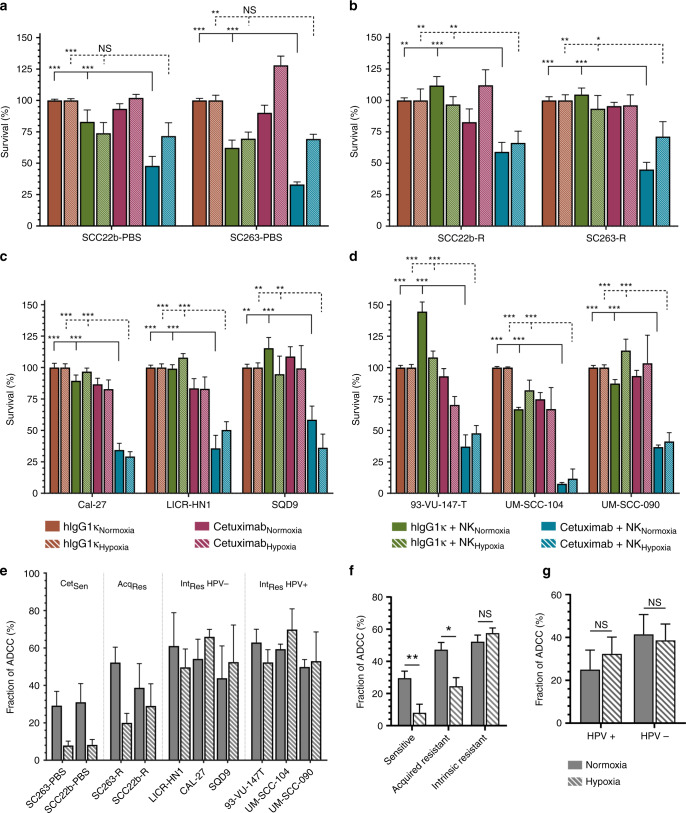
Healthy donor NK-cells combined with cetuximab induce ADCC in HNSCC cell lines. Percentage survival was monitored using the xCELLigence RTCA of cetuximab-sensitive (Cet_Sen_) cell lines (**a**), acquired cetuximab-resistant (Acq_Res_) (**b**) and intrinsically cetuximab-resistant (Int_Res_) cell lines which were either HPV-negative (**c**) or HPV-positive (**d**), until 48 h after treatment. Tumour cells were either treated with an isotype control (IgG1κ) or cetuximab [2 µg/ml], with or without healthy donor-derived NK cells (effector to target (E:T) ratio of 5:1) under both normoxic and hypoxic conditions. Fraction of ADCC induced killing was obtained by subtraction of the means of cetuximab and NK cells from cetuximab alone condition (**e**). Effect of hypoxia was determined within the different cetuximab resistance groups (**f**) and HPV status (**g**) within each group. Graphs represent mean ± SEM for each time point of follow up. Statistical significance was tested using a linear mixed model with Tukey’s pairwise comparison. NS not significant; *p ≤ 0.05; **p ≤ 0.01; ***p ≤ 0.001.

Next, differences in cetuximab-induced ADCC were investigated in our extensive panel of HNSCC cell lines differing in cetuximab sensitivity and HPV status. In addition, the oxygenation state was considered as well. All analyses were performed on the 48-h time point but, in this setting healthy NK cell form blood donors were used instead of the NK92 cell lines. Equal to the aforementioned data, in normoxic conditions, cetuximab [2 µg/ml] alone induced a significant decrease in survival of the Cet_Sen_ cell lines SCC22b-PBS (82.91% ± 9.48) and SC263-PBS (62.23% ± 6.18) compared to isotype control (*p* ≤ 0.001). No decrease in survival was observed in the corresponding isogenic Acq_Res_ clones (*p* > 0.201) (Fig. 3a, b). Similarly, no cytotoxic effect of cetuximab could be detected in the intrinsically cetuximab-resistant cell lines (89.43–144.60 ± 3.14–8.45%;) compared to the isotype control (p ≥ 0.201) except for the UM-SCC-104 (66.92 ± 1.53%; *p* < 0.001) and UM-SCC-090 (87.33 ± 3.18%; *p* = 0.011) cell lines (Fig. 3c, d). In contrast to the NK92 cell line, co-culture of HNSCC cell lines with NK cells from healthy donors did not significantly decrease tumour cell survival (75.35–103.11 ± 2.15–10.49%; *p* ≥ 0.102), except in the UM-SCC-104 (74.86 ± 5.29; *p* < 0.001). However, the addition of cetuximab plus NK cells from healthy donors to HNSCC cell lines induced a statistically significant decrease in cell survival in all ten HNSCC cell lines compared to NK cells alone (16.13–68.90 ± 4.19–20.10%; *p* ≤ 0.007) (Fig. 3a–d).

Under hypoxic conditions, the treatment of Cet_Sen_ cell lines with cetuximab [2 ug/ml] for 48 h showed a reduction in cell survival of 73.940 ± 8.40% and 69.59 ± 5.25% for SCC22b-PBS and SC263-PBS, respectively, compared to isotype control (*p* ≤ 0.004). Neither the Int_Res_ nor Acq_Res_ cell lines showed a significant decrease in survival following cetuximab treatment alone (81.95–113.56 ± 2.82–14.38%; *p* ≥ 0.207). Decrease in survival following co-culturing with NK cells under hypoxia was significant for the 93-VU-147-T and UM-SCC-104 cell lines (67.01–70.27 ± 6.74–17.11%; *p* < 0.001) but not for the other HNSCC cell lines (82.87–127.98 ± 2.84–22.39; *p* ≥ 0.132). Similar to normoxia, addition of cetuximab and healthy donor NK cells to HNSCC cell lines under hypoxia showed a significant induction of ADCC in all cell lines compared to isotype control (11.66–71.67 ± 3.74–11.80%; *p* ≤ 0.009) (Fig. 3c, d).

A multivariate linear mixed model was applied to define the role of cetuximab resistance, HPV status and/or hypoxia on ADCC activity. The fraction of ADCC was calculated through subtraction of the effect of cetuximab monotherapy (Fig. 3e) and used in all further analyses. There was a significant interaction between cetuximab resistance and hypoxia (*p* = 0.002), indicating a non-uniform effect of hypoxia on ADCC activity between the different groups of cell lines. Herein, hypoxia significantly reduced ADCC activity in the Cet_Sen_ (−10.51 ± 3.03%; *p* = 0.004) and the Acq_Res_ cell lines (−11.74 ± 5.29%; *p* = 0.043) but not in the Int_Res_ cell lines (2.05 ± 3.35%; *p* = 0.542) (Fig. 3f). Comparison of the least square means between Cet_Sen_, Int_Res_ and Acq_Res_ cell lines (normoxia: 29.57 ± 4.34%, 52.22 ± 4.09%, 47.31 ± 4.43%; hypoxia: 8.04 ± 5.35%, 57.53 ± 3.21%, 24.51 ± 5.35% respectively) showed a significantly higher ADCC activity in the Int_Res_ resistant cell lines compared to Cet_Sen_ and Acq_Res_ cell lines (*p* ≤ 0.007). Lastly, the influence of HPV status on hypoxia and cetuximab-mediated ADCC was assessed as well (Fig. 3g). No significant interaction between HPV status and hypoxia was observed (*p* = 0.407) and no significant differences in ADCC activity between HPV-positive and HPV-negative cell lines could be detected (*p* = 0.631).

Taken together, statistical analysis suggested that ADCC activity is reduced in the Cet_Sen_ and Acq_Res_ cell lines under hypoxia, while the Int_Res_ cell lines maintain ADCC activity, compared to normoxia. Furthermore, we observed the highest induction of ADCC by healthy NK cells in the Int_Res_ cell lines followed by the Cet_Sen_ and Acq_Res_ cell lines. Finally, HPV status had no significant influence on ADCC activity provoked by cetuximab treatment.

### Cetuximab-mediated ADCC persists irrespective of baseline EGFR surface expression levels

**Fig. 4:**
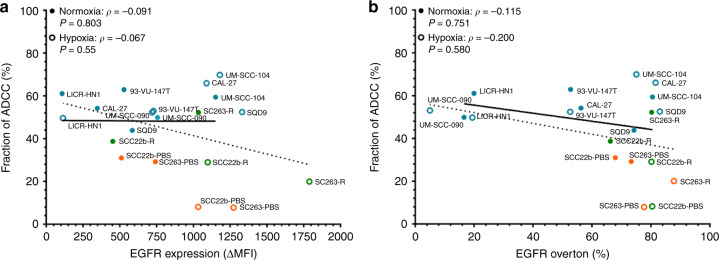
Cell line-dependent EGFR expression on HNSCC cell lines does not determine ADCC. Cell surface levels of EGFR as mean fluorescence intensity (ΔMFI) (**a**) and percentage positivity (overton) (**b**) were correlated with fraction ADCC under normoxia (closed; ●) and hypoxia (open; ○). Correlation coefficients and significance were determined through Spearman's rank-order correlation together with the best fit curve. Points represent the mean of each cell line.

Next, baseline EGFR expression levels were evaluated as a predictor of sensitivity to ADCC. Previously, a cell line-specific expression of EGFR positivity (16.59–80.54 ± 1.90–3.58) and ΔMFI (108.00–1150 ± 23.43–9.21) was observed. These data were shown to be independent of cetuximab sensitivity (overton: *p* ≥ 0.143 and ΔMFI: *p* ≥ 0.170) and HPV status (overton: *p* ≥ 0.302 and ΔMFI: *p* ≥ 0.110).^23^ Under hypoxia, a significant increase in EGFR positivity (4.30–87.12 ± 0.42–16.30; *p* = 0.006) and ΔMFI (124–1797 ± 58.5–581; *p* < 0.001) was observed for most cell lines.^23^ In this study, no correlation was found between EGFR expression and ADCC activity for each cell line (Fig. 4). This was the case both under normoxia (overton: *ρ* = −0.115, *p* = 0.751; ΔMFI: *ρ* = −0.091, *p* = 0.803) and under hypoxia (overton: *ρ* = −0.200, *p* = 0.580; ΔMFI: *ρ* = −0.067, *p* = 0.855). Thus, in our panel of HNSCC cell lines, baseline EGFR expression could not predict ADCC activity.

### Rate of EGFR internalisation determines the degree to which NK cells can induce ADCC

**Fig. 5:**
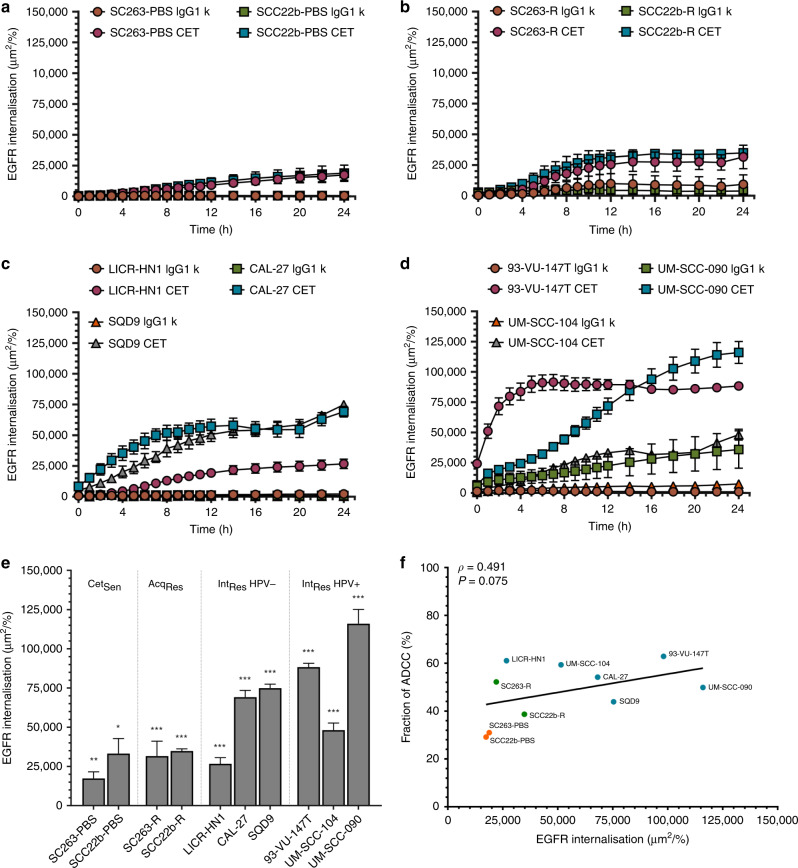
EGFR internalisation kinetics differ in HNSCC cell lines and correlate with ADCC. The amount of EGFR internalisation was monitored using the IncuCyte ZOOM system for the cetuximab-sensitive cell lines (**a**), acquired cetuximab-resistant (**b**) and intrinsically cetuximab-resistant cell lines which were either HPV-negative (**c**) or HPV-positive (**d**). Signal of internalisation at 24 h was compared between cell lines following treatment with cetuximab [2 µg/ml] (**e**). Correlation coefficients and significance were determined through Spearman's rank-order correlation together with the best fit curve (**f**). linear graphs represent mean ± SEM for each time point of follow up and bars represent mean ± SEM at 24 h time point. The total internalisation signal was normalised to confluence of each well. Statistical significance was assessed by one-way ANOVA between ‘Isotype’ and ‘Cetuximab’ treatment conditions. *p < 0.05; **p < 0.01; ***p < 0.001.

Next, we assessed whether internalisation of EGFR could be responsible for the observed differences in ADCC activity between our HNSCC cell lines. To this end, we investigated the amount of EGFR internalisation for each of our cell lines following incubation with cetuximab or isotype control [2 µg/ml] for 24 h. At this time point, cetuximab significantly induced internalisation in all ten cell lines (*p* < 0.001–0.050) compared to the isotype (Fig. 5a–d). All cell lines had reached maximal EGFR internalisation after 12–24 h, as seen by a flattening of the curves (Fig. 5a–d). Therefore, all analyses were performed at 24 h. Furthermore, the amount of EGFR internalisation following cetuximab treatment varied between cell lines. Interestingly, the UM-SCC-090 cell line was the only cell line consistently showing a high degree of internalisation with the isotype (Fig. 5e). EGFR Internalisation following treatment with cetuximab was lowest in the SC263-PBS cell line (17383 ± 4215$\frac{{{\mathrm{\mu m}}^2{\mathrm{/well}}}}{{{\mathrm{\% confluence}}}}$), while the UM-SCC-090 cells displayed the highest EGFR internalisation signal (115 987 ± 9109 $\frac{{{\mathrm{\mu m}}^2{\mathrm{/well}}}}{{{\mathrm{\% confluence}}}}$).

Next, a linear mixed model analysis was performed to test whether differences in EGFR internalisation following cetuximab treatment were due to a difference in cetuximab sensitivity or HPV status. Indeed, there was a clear trend pointing towards a lower degree of internalisation in the Cet_Sen_ cell lines (SCC22b-PBS and SC263-PBS) compared to the Acq_Res_ and Int_Res_ cell lines (*p* = 0.069) (Fig. 5e). In contrast, HPV status did not affect EGFR internalisation (*p* = 0.459). Interestingly, correlating EGFR internalisation with ADCC activity revealed a moderate correlation which showed a statistical trend (*ρ* = 0.491; *p* = 0.075) (Fig. 5f).

Based on these results, we suggest that ADCC activity is independent of EGFR baseline expression. Since both ADCC activity and internalisation were significantly higher in the in Int_Res_ cell lines, together with the positive correlation between both, our results indicate that a higher degree of internalisation might lead to a higher degree of cetuximab-induced ADCC.

## Discussion

The EXTREME trial showed that adding cetuximab to platinum-based CT not only improved response rate (36% vs 20%) but also median OS (10.1 vs 7.4 months) in patients with recurrent/metastatic HNSCC and led to its approval in that setting.^28^ However, absence of durable responses (as a result of the development of resistance) is a limiting factor and a reason why long-term survival figures with this treatment are disappointingly low.^29^ In the present study, we show that cetuximab resistance induces a stronger ADCC response by healthy NK cells in a panel of HNSCC cell lines. Furthermore, we show that regulation of EGFR internalisation might possibly affect the degree of ADCC that can be induced. These findings may be promising for future combinations with immune-oncology drugs.

The present study focused on short-term cetuximab treatment on HNSCC cell lines. Cetuximab treatment of cell lines previously identified as cetuximab sensitive,^23^ had a limited effect on cell survival even at high concentrations. Interestingly, cetuximab treatment of the intrinsically cetuximab-resistant UM-SCC-104 cell line showed a significant decrease in cell survival after 48 h. However, this cell line was still classified as intrinsically resistant based on our previous published work. Herein, we showed that cetuximab treatment of the UM-SCC-104 cell line for 168 or 48 h had a similar decrease in survival.^23^ As opposed to this, 168-h treatment of the cetuximab-sensitive SCC22b-PBS and SC263-PBS cell lines showed a reduction of their mean survival well below 50%. This indicates that after the initial decrease, longer cetuximab treatment of the UM-SCC-104 does not decrease cell survival further and can be considered cetuximab resistant. A possible explanation for the time-dependent cytotoxic effect of cetuximab could be, as suggested by Narvi et al., that initial/short-term treatment with cetuximab mostly promotes cell cycle arrest, while a sustained inhibition of EFGR signalling initiates apoptotic signalling pathways.^30^

Besides inhibition of EGFR signalling, cetuximab binds Fc receptors on NK cells.^17^ Interestingly, in comparison to other immune infiltrated tumour types, HNSCC tumours are marked by the highest levels of NK cell infiltration,^31^ showing the importance of NK cells in further investigations. Previous studies have demonstrated the ability of cetuximab to induce ADCC in various tumour types.^17,32,33^ Furthermore, inhibition of anti-inflammatory cytokines, such as TGF-β, or addition of pro-inflammatory cytokines, such as IL-2 and IL-15, enhanced cetuximab-mediated ADCC.^34^ However, knowledge regarding the effect of cetuximab sensitivity and/or HPV status on ADCC activity remains scarce. Therefore, in the present study we focused on determining ADCC activity of cetuximab in a panel of HNSCC cell lines with different cetuximab sensitivities and HPV status under both normoxic and hypoxic conditions. Addition of cetuximab to co-cultures of HNSCC cells with NK cells clearly induced ADCC in all cell lines. However, the extent of ADCC induced differed significantly among cell lines. In this regard, our study is the first to show that cell lines with acquired resistance against cetuximab are equally susceptible to cetuximab-mediated ADCC as their corresponding isogenic cetuximab sensitive cell lines. Surprisingly, intrinsically cetuximab-resistant cell lines were more susceptible to ADCC compared to sensitive and acquired resistant cell lines in our assay.

Considerable research has tried to elucidate cetuximab resistance mechanisms.^35^ However, research investigating the possible effect of cetuximab sensitivity on ADCC induction has been limited. One mechanism described is the presence of an EGFR-K_521_ polymorphism, shown by Braig et al., which reduced the effectiveness of cetuximab binding to EGFR and thus reduced EGFR signalling and ADCC.^36^ To the best of our knowledge, the study of Braig et al. is the first to emphasise the unique immune-related effect of cetuximab resistance mechanisms.

We investigated two additional potential mechanisms, to unravel the complex interplay between ADCC and cetuximab sensitivity. First, we hypothesised that expression levels of EGFR might determine ADCC activity. However, we found no significant correlation between cetuximab-mediated ADCC and EGFR expression, both under normoxia and hypoxia. Other in vitro studies report varying results. While some studies do not observe a correlation,^37^ others describe a correlation between EGFR expression and cetuximab-mediated ADCC.^32,38^ Clinical reports show discordant results as well^39,40^ and therefore EGFR expression is currently not a recognised predictive biomarker for cetuximab treatment in HNSCC. Similar results were observed for panitumumab, an IgG2 mAb against EGFR which functions exclusively through receptor inhibition.^41^ Together with our results, this suggests that (i) even low EGFR expression levels may be sufficient to induce maximal ADCC by NK cells, and (ii) the involvement of other biological mechanisms might be the cause for the discrepancy in literature and the difference in ADCC among our cell lines.

Second, to further investigate this matter, we determined internalisation of EGFR following treatment with cetuximab. Herein, one would expect a reduction in surface expression of EGFR to decrease the possibility of recognition by cetuximab and subsequently induction of ADCC as well. In our results, we showed that levels of EGFR internalisation varied between HNSCC cell lines and found a positive correlation between EGFR internalisation and ADCC. While receptor internalisation is known as a signal attenuation process, the idea that internalisation is required for activation of specific downstream transcription molecules is gaining interest.^42^ Interestingly, in contrast to ligand-bound EGFR internalisation, cetuximab-bound EGFR has an increased stability under endosomal pH levels and therefore is targeted for lysosomal degradation rather than recycling or trafficking to other cell organelles.^43^ Thus, binding of cetuximab leads to downregulation of EGFR, reducing the amount of available surface EGFR for ADCC induction. However, based on our data and those of Kol et al.,^33^ internalisation per se was not sufficient to impair ADCC induction. Therefore, we postulate that internalisation of EGFR is able to reduce—but not abolish—surface expression of EGFR. This has also been shown in colorectal cancer,^44^ and suggests that a minimal threshold for induction of ADCC exists and that this threshold is maintained following treatment with cetuximab. Moreover, our results showed that internalisation even increased ADCC effects. Further research investigating the mechanisms of internalisation within cell lines with different cetuximab sensitivities is necessary to improve therapeutic approaches considering NK cell-mediated ADCC through cetuximab.

Besides cetuximab resistance, we also investigated the role of hypoxia on ADCC. Previous studies have shown that hypoxia affects the functionality of NK cells. Both expression of the NKG2D and, albeit to a lesser extent, CD16 receptors as well as intracellular perforin and granzyme B were decreased under hypoxia.^21,45^ Others showed that levels of CD16 expression do not correlate with the intensity of ADCC,^46^ suggesting that hypoxia could abolish the natural cytotoxicity of NK cells but not ADCC. Our results are in line with these reports, showing that ADCC is maintained under hypoxia. However, while hypoxia did not affect ADCC sensitivity of acquired and intrinsic resistant cell lines, cetuximab-sensitive cell lines did become more resistant to NK cell-mediated ADCC. In this regard, hypoxia is known to modulate both proteomic and genomic changes within tumour cells.^47^ Our results suggest that modulation of these processes is different between cell lines with different cetuximab sensitivities and thereby differently influences susceptibility for ADCC. However, further research is warranted to elucidate the mechanisms behind the reduced ADCC within our cetuximab-sensitive cell lines compared to the acquired resistant and intrinsic resistant cell lines under hypoxia.

Finally, although HPV is a known positive prognostic factor in HNSCC,^48^ ADCC activity in HPV-positive cell lines was comparable to HPV-negative cell lines in our setting, showing only a statistical trend. Future studies with isogenic HPV-positive and -negative cell lines could provide more insight into the role of HPV. However, similarly to our results, a recent study in cetuximab-treated recurrent or metastatic HNSCC patients did not observe a relationship between HPV status and ADCC inducibility despite observing a better OS.^15^ In this regard, the prognostic benefit of HPV is suggested to be a result of a pre-existing immune response against HPV-positive cells.^49^ Studies have shown that HPV-positive HNSCC were associated with a higher number of intratumoural NK cells,^50^ which may partly explain the increased OS. However, as shown by Taylor et al., there was no difference in ADCC between HPV groups, taken into account the number of NK cells.^15^ Therefore, these results suggest that NK cells play a major role against HNSCC and that, in contrast to an increased number of NK cells, HPV status does not affect their activity. However, the mutational profile and molecular characteristics between HPV-positive and -negative HNSCC have been shown to differ as well, adding to the heterogeneous complexity of HNSCC and disparities in therapeutic responses.^51^

## Conclusions

In conclusion, our study presents novel insight into the impact of cetuximab resistance, HPV status and hypoxia on the efficiency of cetuximab-based tumour cell killing. We found that mechanisms of cetuximab resistance may enhance effector cell-based killing. Further research into these mechanisms could help overcome cetuximab resistance by combining immunotherapies with targeted therapies. Furthermore, we show that induction of ADCC is maintained, independent of surface EGFR expression levels and that internalisation of EGFR is closely linked with resistance to cetuximab. Further research into unravelling this mechanism will provide a better understanding of cetuximab-based therapies in the future.

## Supplementary information

Supplemental material

## Data Availability

All data generated or analysed during this study are available from the corresponding author on reasonable request.
