# Supplementary material for: Cetuximab-induced natural killer cell cytotoxicity in head and neck squamous cell carcinoma cell lines: investigation of the role of cetuximab sensitivity and HPV status
Source: Br J Cancer. 2020 Jun 16;123(5):752–61. doi: 10.1038/s41416-020-0934-3 (PMC7462851; doi:10.1038/s41416-020-0934-3)
Supplement: Supplementary file 1 — Supplemental material [file 41416_2020_934_MOESM1_ESM.pdf]

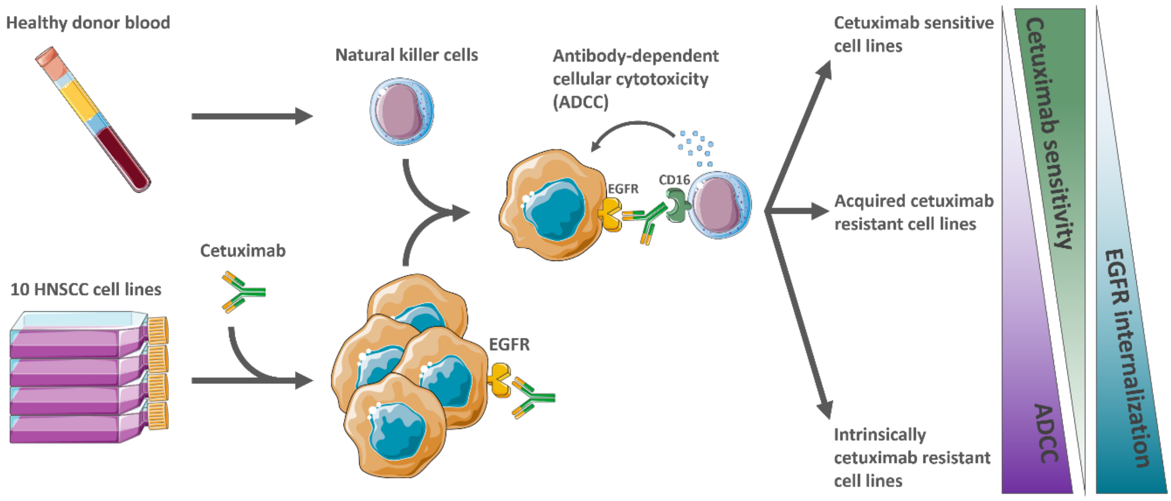

Figure S1 Graphical abstract representing the experimental flow. A panel of HNSCC cell lines were grouped according to their cetuximab sensitivity. Co-culture with healthy blood derived NK cells in addition to cetuximab showed differential ADCC induction between cell lines. Correlations between ADCC, cetuximab sensitivity and EGFR internalization were found.

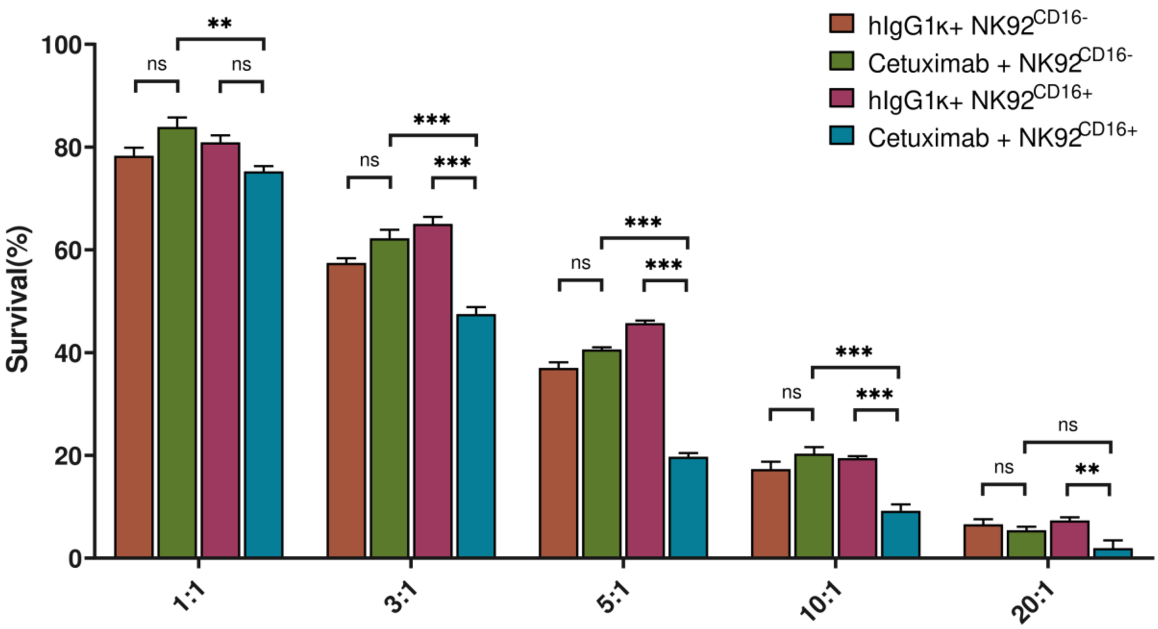

Figure S2 Effector to Target (E:T) ratio-dependent effect of NK92 cell lines (CD16+/-) on the survival of the SCC22b-PBS HNSCC cell line. Percentage survival of the SCC22b-PBS was

measured using the xCELLigence RTCA. Following overnight incubation, HNSCC cell lines were treated with an isotype control (hIgG1κ) or cetuximab (2µg/ml). NK92 cells were added in several effector to target (E:T) ratios, and survival was monitored for 48h post-treatment. Both CD16-positive and CD16-negative NK92 cells were used. Graphs represent mean ± SEM for each time point of follow up. Statistical significance was tested with Tukey's pairwise comparison. NS= not significant; *=$p<0.05$; **=$p<0.01$; ***= $p<0.001$.
